# Detection of Human Papillomaviruses by Polymerase Chain Reaction and Ligation Reaction on Universal Microarray

**DOI:** 10.1371/journal.pone.0034211

**Published:** 2012-03-23

**Authors:** Jarmo Ritari, Jenni Hultman, Rita Fingerroos, Jussi Tarkkanen, Janne Pullat, Lars Paulin, Niina Kivi, Petri Auvinen, Eeva Auvinen

**Affiliations:** 1 Institute of Biotechnology, University of Helsinki, Helsinki, Finland; 2 Department of Virology, Helsinki University Hospital Laboratory, Helsinki, Finland; 3 Department of Virology, Haartman Institute, University of Helsinki, Helsinki, Finland; 4 Department of Pathology, Haartman Institute, University of Helsinki, Helsinki, Finland; 5 Functional Genome Analysis, Deutsches Krebsforschungszentrum, Heidelberg, Germany; National Institute of Health - National Cancer Institute, United States of America

## Abstract

Sensitive and specific detection of human papillomaviruses (HPV) in cervical samples is a useful tool for the early diagnosis of epithelial neoplasia and anogenital lesions. Recent studies support the feasibility of HPV DNA testing instead of cytology (Pap smear) as a primary test in population screening for cervical cancer. This is likely to be an option in the near future in many countries, and it would increase the efficiency of screening for cervical abnormalities. We present here a microarray test for the detection and typing of 15 most important high-risk HPV types and two low risk types. The method is based on type specific multiplex PCR amplification of the L1 viral genomic region followed by ligation detection reaction where two specific ssDNA probes, one containing a fluorescent label and the other a flanking ZipCode sequence, are joined by enzymatic ligation in the presence of the correct HPV PCR product. Human beta-globin is amplified in the same reaction to control for sample quality and adequacy. The genotyping capacity of our approach was evaluated against Linear Array test using cervical samples collected in transport medium. Altogether 14 out of 15 valid samples (93%) gave concordant results between our test and Linear Array. One sample was HPV56 positive in our test and high-risk positive in Hybrid Capture 2 but remained negative in Linear Array. The preliminary results suggest that our test has accurate multiple HPV genotyping capability with the additional advantages of generic detection format, and potential for high-throughput screening.

## Introduction

Human papillomavirus (HPV) infects mucosal and cutaneous epithelia and is one of the most common sexually transmitted infections. Currently there are over 120 known HPV genotypes [1], [2] associated with a number of diseases including many cancers, particularly cervical cancer. While HPV infection is the cause of essentially all cases of cervical premalignant lesions and cervical cancer [3], [4], most infections even with high-risk HPV (hrHPV) types are transient and resolve without causing disease [5]. Persistent high-risk infections among women more than 35 years of age in contrast have much higher probability of developing into cervical precancer and eventually progression to invasive cancer [6]. Primary screening using HPV DNA testing with cytology triage has been shown to be more sensitive and, importantly, more specific than conventional testing based on cytology among women more than 35 years of age [7]–[9]. The nearly 100% negative predictive value of hrHPV testing would allow returning hrHPV negative patients to the normal screening program and even enable longer screening intervals for the general population [10]. Regular population screenings have significantly decreased deaths from cervical cancer in the developed world, but it is still one of the most common malignancies worldwide [11]. As hrHPVs, especially HPV types 16 and 18 in comparison to other hrHPVs, differ in their clinical behavior, HPV genotyping could help increase the positive predictive value of HPV testing. Accurate risk prediction is likewise valuable in clinical surveillance and management of patients after colposcopy.

HPV testing is currently largely based on either direct liquid hybridization or PCR amplification of polymorphic L1 or E1/E6/E7 viral genomic region using consensus or multiplex primers. The current standard non-PCR molecular test, the Hybrid Capture 2 assay, is based on liquid hybridization of RNA probes to target DNA followed by antibody detection. It is able to differentiate between low-risk and high-risk HPV infections but cannot determine specific HPV genotypes. The Linear Array HPV Genotyping test is capable of differentiating 37 HPV types but is laborious and has limited throughput capacity. The demand for high-throughput genotyping has motivated the application of oligonucleotide DNA microarray technology [12] to HPV detection and identification by several groups [13]–[16]. In this approach, oligonucleotide probes immobilized on microarray capture fluorescently labeled HPV PCR amplicons and are reported to successfully discriminate between HPV types. Thus, DNA microarray has the potential for a screening tool in clinical settings. However, currently there is no standard for HPV multiplex PCR and published primer sets vary in their ability to amplify multiple HPV templates [17]–[20].

Here we report a highly sensitive and specific DNA microarray method for genotyping of 15 different hrHPVs and two low-risk HPVs (lrHPVs). The method is based on multiplex PCR amplification of the viral L1 region using an improved set of type-specific PGMY primers (modified from [21]) and subsequent ligation detection reaction (LDR). Further, we establish an objective criterion for HPV type presence by statistical comparison to a background distribution. The ligation method relies on the high selectivity of a ligase enzyme which requires complementarity of the 3′ structure to successfully catalyze the covalent joining of two adjacently hybridized ssDNA probes [22], [23]. The probes constitute a target-specific probe pair, which becomes detectable only if the probes are ligated together in the presence of target. The 5′ labeled discriminating probe is designed such that the 3′-end matches the target at a unique position that distinguishes the target from other known DNA targets. The 5′ phosphorylated common probe, flanking a unique 3′ ZipCode sequence, is designed to hybridize adjacent to the discriminating probe, thus enabling ligation only when an appropriate target is present in the reaction mixture. The ligation products are detected on a low-density universal microarray harboring complementary Zip sequences (cZipCodes) [24]. The ligation method has been shown to be able to accurately profile complex microbial populations ([25]–[27]). We applied LDR and universal microarray method to the specific detection and identification of high risk HPV types from patient samples after testing the platform with different HPV types alone and in a mixture.

## Materials and Methods

### Ethics Statement

The use of human samples was approved by the ethics committees at all hospitals where participants were recruited and human samples were collected: the Coordinating Ethics Committee of the Hospital District of Helsinki and Uusimaa (69/E0/2007) and the National Supervisory Authority for Welfare and Health (2461/04/044/07). A written informed consent was signed by all patients participating in the study. All data were analyzed anonymously.

### HPV plasmids

Cloned plasmids of full-length HPV 6, 11, 16, 18, 31, 33, 35, 39, 42, 43, 44, 51, 52, 56, and 58 were generously provided by Drs E.-M. de Villiers, W. Lancaster, A. Lorincz, T. Matsukura, G. Orth and S. Silverstein. Additionally, the PCR target sequences of HPV types 45, 59, 68, 73, and 82 were synthesized and cloned into the pCR2.1 vector (Eurofins MWG Operon, Ebersberg, Germany).

### Patient samples

HPV samples sent to the Department of Virology for laboratory diagnostics of HPV infections using the Hybrid Capture 2 test (HC2; Qiagen, Gaithersburg, MD) were used in the study.

### DNA extraction

Extraction of DNA from patient samples on the MagNAPure LC instrument (Roche Applied Science, Indianapolis, IN) was optimized for HC2 samples. The MagNA Pure LC Total Nucleic Acid Isolation Kit (Roche Applied Science, Indianapolis, IN) turned out to be optimal. For each nucleic acid extraction, 50 µl of HC2 sample was used. For samples tested with Linear Array, 200 µl of HC2 sample was used.

### Hybrid Capture 2 and Linear Array tests

The HC2 test kit for hrHPV was purchased from Qiagen (Gaithersburg, MD) and was used according to manufacturer's instructions. The LA test kit was purchased from Roche (Basel, Switzerland) and was used according to manufacturer's instructions.

### PCR amplification

HPV plasmids and patient samples were amplified with Qiagen Multiplex PCR kit in a 25 µl reaction volume containing 12.5 µl of 2× Master Mix, 5 pmol of each type-specific PGMY-t primer and 1 ng of plasmid DNA or 8.5 µl of extracted patient sample DNA. In each PCR reaction using HPV plasmid template, forty nanograms of extracted DNA from HPV-negative human placenta was added to mimic an environment containing chromosomal DNA. Amplifications were performed in a thermal cycler (MJ Research, MA) starting with polymerase activation at 95°C for 10 min and 40 cycles of 95°C for 1 min, 55°C for 1 min and 72°C for 1 min followed by a final extension at 72°C for 10 min. The amplification reaction mixtures were stored at 4°C. Five-µl aliquots were visualized in agarose gels, and 20 µl were purified using the PCR Purification Kit (Qiagen, Hilden, Germany) and eluted in 30 µl distilled water. The PGMY-t HPV multiplex primer mix sequences are listed in File S1. The PCR primers for human beta-globin gene were 5′-GAAGAGCCAAGGACAGGTAC-3′ (GH20; forward) and 5′-CAACTTCATCCACGTTCACC-3′ (PC04; reverse) [28]. The PGMY-t primer mix was tested by dividing the mix into four pools of five primer pairs each in order to assess the multiplexing capacity. The five low-risk HPV types 6, 11, 42, 43 and 44 were pooled into one pool (lr-PGMY-t). The 15 high-risk HPV types were divided into three different pools so that each pool contained five hr-HPV types which were phylogenetically as distant from each other as possible. The pools were as follows: HPV 16, 18, 33, 39, 51 (hr1-PGMY-t); HPV 31, 45, 52, 68, 82 (hr2-PGMY-t); HPV 35, 56, 58, 59, 73 (hr3-PGMY-t). The plasmids were pooled in a similar manner and 40 pg/µl DNA extracted from human placenta testing HPV negative was added in each pool. The PCR fragments were sequenced in the DNA sequencing service at the Institute of Biotechnology, University of Helsinki.

### Ligation probe design

The viral genome sequences obtained from NCBI database were managed using Staden Package Gap 4 v4.8b1 software [29]. Ligation probe pairs, each consisting of a discriminating probe and a common probe, were designed to target the variable L1 genomic region amplified using the MY primers. Target specificity of the selected probe candidates was verified using BLAST [30]. Two probe pairs were designed for each HPV type and one pair for the human beta-globin gene. The T_m_ for each discriminating probe and common probe was set to approximately 55°C as computed by OligoCalc Nearest Neighbor algorithm [31]. The ZipCode oligo sequences flanking each common probe were selected from the Affymetrix GenFlex Tag Array (Affymetrix, Santa Clara, CA) data file downloaded from the Affymetrix website. The Cy3-labeled discriminating oligos and phosphorylated common oligos were synthesized by Oligomer Oy (Helsinki, Finland). The ligation probe and ZipCode sequences are listed in File S1.

### Ligation reaction

**Figure 1:**
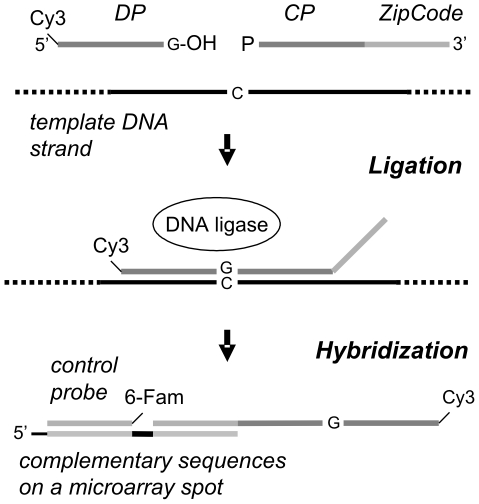
The principle of ligation detection reaction. The discriminating probe (DP) and the common probe (CP) are designed to hybridize adjacently on the template DNA and are joined by ligase in the presence of a matching template (HPV PCR product). The discriminating 3′ base can be A, C, T or G. The reaction is thermally cycled and ligation products addressed on microarray spots by the unique ZipCode sequences flanking each CP. Hybridization control probe carries a different label (6-Fam) than the DP (Cy3).

The LDR (Figure 1) was carried out in a final volume of 20 µl containing 1× ligation buffer (Taq ligase buffer, New England Biolabs, MA), 30 mM tetramethylammonium chloride (TMAC; Sigma-Aldrich, Steinheim, Germany), 250 fmol of each discriminating probe, 250 fmol of each common probe, 2 µl of sample PCR product or 40 fmol of plasmid PCR product, and 5 U Taq DNA ligase (New England Biolabs, MA). The reaction was cycled for 40 rounds at 94°C for 30 s and at 55°C for 4 min in a thermal cycler (MJ Research, MA).

### Microarray fabrication

The 16 compartment microarray slides were spotted with 119 different complementary ZipCode oligonucleotides (synthesized by Bioneer, Daejeon, South Korea) in triplicate. Each cZipCode oligo also contained a sequence complementary to the hybridization control probe (5′-TCAATGCACTGAGCCCGAGA-3′) [32]. The amine coated SuperMask16 slide substrates and printing were both purchased from Arrayit (Sunnyvale, CA). The 8×15K format custom synthesized oligonucleotide microarrays were purchased from Agilent (Santa Clara, CA). The microarray was designed using Agilent's eArray web tool. HPV LDR probe specific oligonucleotide sequences on the microarrays are listed in File S1.

### Microarray hybridization and scanning

The ligation reaction (20 µl) was diluted to obtain 40 µl of hybridization mixture containing 5X SSC and 10 µg of herring sperm DNA. After heating the mixture to 94°C for 2 min and chilling on ice, 5 pmol of the complementary hybridization control probe was added and the mixture was applied onto the microarray slide according to manufacturer's instructions. Hybridization was carried out in the dark at 55°C for two hours in a temperature controlled hybridization oven. After hybridization, the microarray was washed for 3×15 min in 0,1× SSC, 0.1% SDS and 3×5 min in distilled water. Finally, the slide was dried in a table top centrifuge. For Agilent microarrays, hybridization was performed according to the manufacturer's instructions. Briefly, hybridization mixture containing 1× GEx hybridization buffer (Agilent Technologies, Cedar Creek, TX), 1× GEx blocking reagent (Agilent Technologies, Cedar Creek, TX), 18 µl of ligation reaction and 1 µl (5 pmol) of control oligo was applied on each subarray and hybridized for 17 h in the dark at 65°C at 10 rpm rotation. The slide was washed with Gene Expression wash buffer 1 (Agilent Technologies, Wilmington, DE) for 1 min at RT and Gene Expression wash buffer 2 (Agilent Technologies, Wilmington, DE) for 1 min at 37°C. 10% Triton X-102 was added to both washing solutions to final concentration of 0.005%. The fluorescent signal was detected at 5 or 10 µm resolution using a GenePix Autoloader 4200AL laser scanning system with green laser for Cy3 dye (abs 532 nm/em 550 nm, LDR-probes) and blue laser for 6-FAM (abs 488/em 495, hybridization control channel) [25], [33]. Both the laser and the photomultiplier (PMT) tube power were set at 100%. GenePix program version 6.1 was used to quantitate the fluorescent signal from each microarray spot. The microarray data are MIAME compliant and have been deposited in ArrayExpress database (accession numbers E-MEXP-3152, E-MEXP-3153, E-MEXP-3154, E-MEXP-3155, E-MEXP-3466, E-MEXP-3477).

### Microarray data processing

The microarray data were managed using R-software [34] and Bioconductor package *marray* [35]. The microarray raw signals were normalized as described previously [32]. Briefly, after local background subtraction, the control channel values were multiplied by the ratio of medians of probe channel and control channel. Next, negative values were removed and probe channel signals were adjusted as *L* ^′^_i_ = *L*_i_log(*L*_i_/*C*_i_), where *L*_i_ is the raw probe channel signal value at feature *i* and *C*i is the adjusted control channel signal value at feature *i*.

### Signal classification

The background signal distribution in HPV detection was determined by selecting the normalized signals of all non-specific probes for each template from each microarray used in the type specificity experiments and excluding signal values over 2000. The background signal distribution served as a reference against which probe signals were compared with one-sided statistical testing. The background distribution was adjusted for each individual microarray hybridization by computing the difference between the background distribution median and the median of three highest values in known negative probes (non-functional probes; A84, A106, A87, A88, A89, A90, A111). Three one-sided statistics, t-test with unequal variances (Welch's test) and two non-parametric tests (Mann-Whitney and Kolmogorov-Smirnov), were evaluated in their ability to differentiate true positive (templates present in mixture; A99, A117, A101, A119, A102, A120, A59, A83, A112) and true negative (functional probes but templates not present in mixture; A100, A103, A104, A105, A107, A108, A109, A110, A113, A114, A115, A118, A29, A53, A54, A55, A56, A57, A58, A85, A25, A86, A91) signals from 23 replicated microarrays. The performance of the tests was evaluated by ROC with p-values between 0 and 1 and by computing the overlap of p-value distributions. Mann-Whitney with p-value limit of 0.005 was used in final testing.

## Results

### Evaluation of target specificity of the ligation probe pool

**Figure 2:**
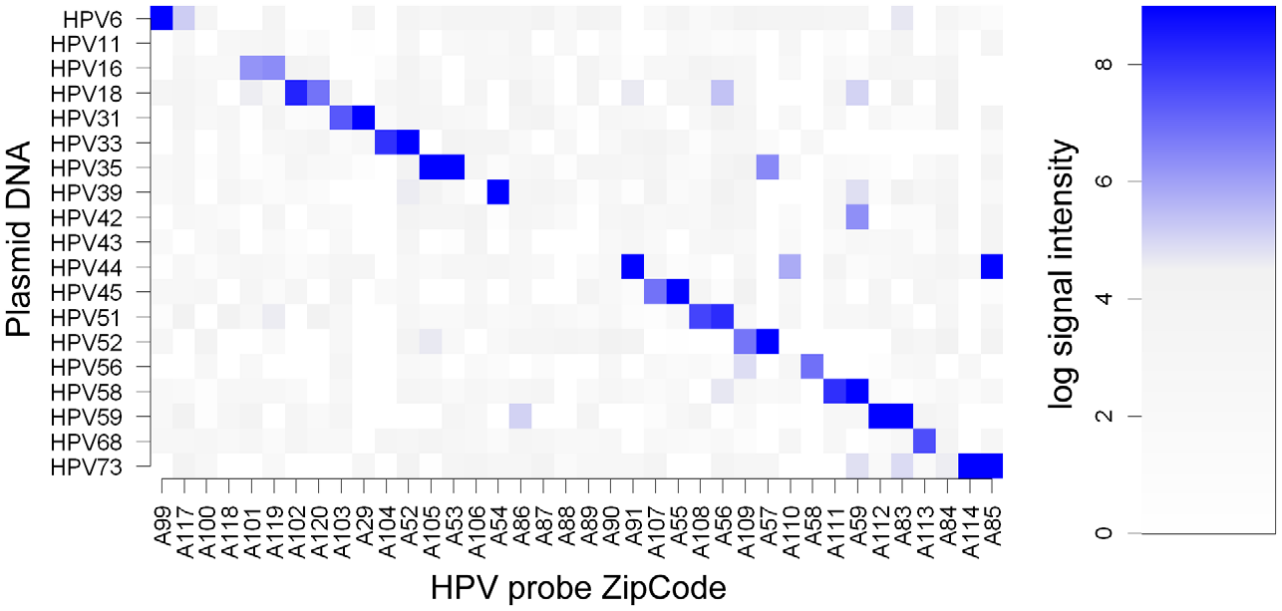
Specificity of HPV LDR probe pool. The HPV LDR probe pool was tested against individual plasmid templates. The horizontal axis shows the probes by ZipCodes and corresponding HPV types. The vertical axis shows plasmid templates by HPV type. The signals are medians over spot replicates on 1–3 microarrays. The majority of the probes give a high hybridization signal only for their specific target plasmids. For HPV 11, 42 and 43, there are no functional probes. HPVs 39, 56 and 68 have only one functional probe each. The probe A85 (HPV 73 ) shows a strong nonspecific hybridization signal to HPV 44 template. Minor nonspecific hybridisation signals are evident in probes A59, A110, A57 and A56.

In order to establish functionality and type specificity of the test, we prepared ligation reactions for each HPV type individually using 40 fmol HPV plasmid PCR products as templates for the ligation probe pool. The majority of the probes produced specific signals on their cognate templates easily distinguishable from the background of non-specific probe signals (Figure 2). However, thirteen out of 38 HPV probes turned out not to be properly functional, resulting in three HPV types (11, 42, 43) not having any functional probes, and four HPV types (6, 39, 56, 44) each having only one functional probe in the pool (Figure 2). The probe A85 (HPV 73) gave a strong nonspecific signal for HPV 44 plasmid while probes A110 (HPV 56), A57 (HPV 52), A86 (HPV 42) and A56 (HPV 51) gave moderate signals to one non-target plasmid each. The probe A59 (HPV 58) was positive for two non-target plasmids. In total, out of 684 (18×19×2) possible nonspecific probe-template combinations, 8 signals were considered false positives. The internal control probe for endogenous human beta-globin was occasionally negative when a strong positive signal was obtained for HPV.

### Testing of multiplex PCR primer mixes

**Figure 3:**
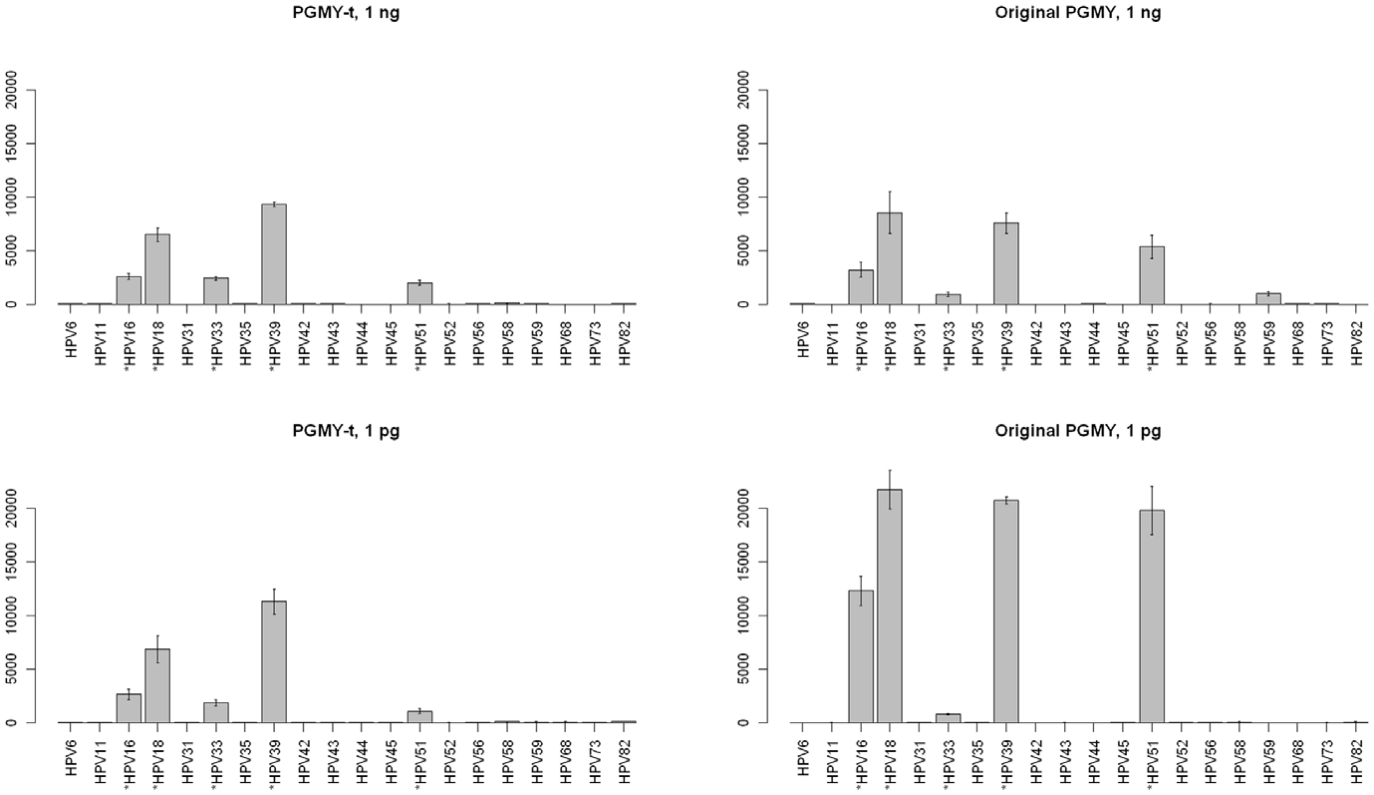
Comparison of the PGMY-t and the original PGMY multiplex PCR primer mixes as measured by HPV LDR signals on microarray. Both primer mixes at 0.2 µM primer concentration amplify all five HPV types from 1 ng of template (A) & (B) and from 1 pg of template (C) & (D). With the original PGMY mix at 1 ng template concentration, HPV 59 gives a false positive signal (B). HPV 33 signal is also relatively weak with the original PGMY at 1 pg template concentrations (D). Data are presented as means±SD from two independent microarrays. Y-axis shows signal intensity in arbitrary units. Asterisk (*) indicates the target HPV types present in the experiment.

After initial testing of MY09/11, GP5+/6+ [36] and PGMY primers [37] we selected to use the PGMY which were capable of more efficient and uniform amplification of different HPV types. However, after extensive optimization of PGMY PCR conditions, the multiplexing capacity of these primers still remained inefficient. Finally, we synthesized PGMY primer pairs specific and 100% identical to each of the desired HPV types (PGMY-t primer mix). The performances of the PGMY-t mix, hr1-PGMY-t mix and the original PGMY mix were assessed by amplifying high-risk pool 1 plasmids (containing HPVs 16, 18, 33, 39, 51) and using the PCR products as templates for HPV ligation reactions. The original PGMY primer mix amplified HPV 33 from hr1 plasmid pool relatively inefficiently. Further, it gave an unspecific signal with the HPV 59 at higher template concentrations of 1 ng and 100 pg (Figure 3; File S4). The PGMY-t mix performance was approximately similar to that of hr1-PGMY-t mix in being able to amplify all correct templates without producing false positive signals at 1 pg −1 ng template concentrations with both 0.1 and 0.2 µM primer concentrations (Figure 3; File S4). In each multiplex PCR reaction with plasmid/placenta or DNA from human samples, a fragment of the gene coding for human beta-globin was coamplified together with HPV using the PC04 and GH20 primers. Test evaluation for human samples was done using 0.2 µM PGMY-t primer mix.

### Sensitivity

In order to determine the sensitivity of the method, we performed a dilution series experiment using HPV 16 and 18 plasmids as templates for PGMY-t HPV PCR. The plasmid amount ranged from 1 pg to 0.1 fg. The amplicons were detected by HPV LDR probes on microarray. One fg was the lowest detectable amount of plasmid, after which the signals from HPV 16 and 18 probes were indistinguishable from other HPV LDR probes (File S5). Signals from 1 fg plasmid concentration were slightly higher than from 1000 fg concentration.

### Differentiation of positive and negative signals

**Figure 4:**
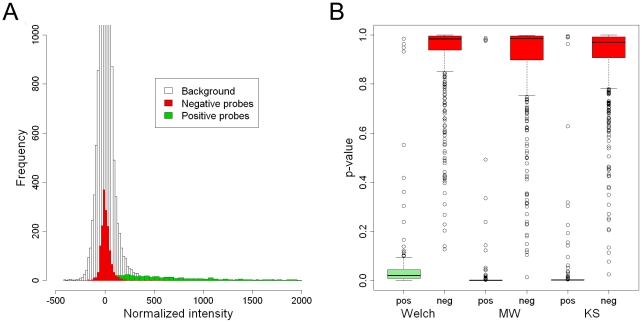
Evaluation of positive and negative microarray signals. A) Distributions of background signals and signals from true positive probes (green) and true negative probes (red). For clarity, frequencies above 1000 and tails of intensity distributions over 2000 are not shown. B) P-value distributions of three statistical tests. Positive (pos) and negative (neg) probes are tested against the background distribution.

In order to classify signals into positive and negative categories, we compared three statistics in their ability to differentiate true positive and true negative signals. The signals from the background and true negatives distributed similarly close to zero, as expected, and the true positives distributed more widely partially overlapping with the background (Figure 4A). All three statistical tests performed well with basically no differences as estimated by receiver operating characteristic (ROC) which plots sensitivity vs. 1-specificity at different classifier parameter values, i.e. in this case p-values ranging from 0 to 1 (not shown). However, plots of the p-value distributions show that Welch's test tends to give clearly higher p-values for positive signals than the non-parametric tests (Figure 4B). Only approximately 78% of true positive signals had p-value below 0.05 with Welch's test while for Mann-Whitney and Kolmogorov-Smirnov the percentages were 95 and 93, respectively. Mann-Whitney and Kolmogorov-Smirnov p-values, 90% and 85% respectively, were below 0.005 whereas only 17% of Welch p-values were below this limit. Based on the results, we chose Mann-Whitney test to be used in the final microarray data analysis. A HPV probe signal was classified as positive if the obtained p-value was less than 0.005. As an additional criterion of test validity, we performed co-amplification of human beta-globin in order to control the sample adequacy and quality, as well as success in DNA extraction and multiplex PCR (internal control). Microarrays having both negative HPV and beta-globin were discarded as this indicates that the sample is inadequate or inhibitory, or that PCR or any other step thereafter has not worked optimally.

### Evaluation of the LDR test using clinical samples

Two sets of patient samples were tested in order to evaluate the performance of LDR. First, a set of 16 samples was tested with LDR and the widely used non-genotyping Hybrid Capture 2 test (HC2) (Table 1). Sample p403/2z was tested in triplicate (File S2) and sample p411/11z in quadruplicate, respectively, to evaluate assay reproducibility. Out of 16 patient samples, 12 were hrHPV positive, one was lrHPV positive and three remained negative in the LDR test (Table 1). Seven samples were hrHPV positive when tested with HC2. Ouf of the 12 hrHPV positive samples in LDR, five were negative in HC2. All samples negative in LDR were also negative in HC2. In all, 10 out of 16 samples were concordant between LDR and HC2 (63%). Multiple HPV types were found by LDR in five samples. Identical HPV types were identified in all replicates. In addition, the PCR products of the two patient samples done in replicates were sequenced, confirming the presence of those HPV types detected by LDR (File S3). A sample from human placenta (Table 1) was HPV negative in both LDR and HC2. Second, using another set of 16 samples the performance of LDR was compared to HC2 and the genotyping LA (Table 2). Testing of one patient sample (sample 9; PO1075) by LA was invalid, because the betaglobin control bands failed to show (File S6). Altogether 14 out of 15 valid samples (93%) gave concordant results. Sample 13 (PO1084) was HPV 56 positive in LDR and HC2 whereas it remained negative in LA. There were minor differences in genotyping results between LA and LDR. In sample 2 (PO1065) LA detected HPV 83 which is not included in our test. In sample 7 (PO1070) LA detected HPV type 70, and in sample 12 (PO1082) HPV 62 was found, which were not covered by our test (Table 2). The LDR signals for the 16 patient samples in Table 2 are presented in File S7.

**Table 1:** Analysis of patient samples with LDR and Hybrid Capture 2.

Sample	Type	LDR	HC2
PO145	patient	HPV 18	neg
PO146	patient	HPV6	neg
PO147	patient	-	neg
PO148	patient	HPV 18 16, 73	neg
PO149	patient	HPV 18 33	neg
PO150	patient	HPV 31	pos
PO151	patient	-	neg
PO152	patient	HPV 16	neg
PO153	patient	HPV 16, 82	neg
PO154	patient	HPV 16	pos
PO155	patient	HPV 33, 35	pos
PO156	patient	HPV 18	pos
PO157	patient	HPV 33	pos
PO159	patient	-	neg
p403/2z*)	patient	HPV 16, 31, 33, 45	pos
p403/2z*)	patient	HPV 16, 31, 33, 45	pos
p403/2z*)	patient	HPV 16, 31, 33, 45	pos
p411/11z*)	patient	HPV 51	pos
p411/11z*)	patient	HPV 51	pos
p411/11z*)	patient	HPV 51	pos
p411/11z*)	patient	HPV 51	pos
placenta	placenta	-	neg

*) Sequenced samples.

**Table 2:** Analysis of patient samples with Roche Linear Array, LDR and Qiagen Hybrid Capture 2.

Sample	Linear Array	LDR	HC2
1 (PO1064)	16	16	pos
2 (PO1065)	18, 45, 51, 83	18, 45, 51	pos
3 (PO1066)	52/33/35/58, 58	58	pos
4 (PO1067)	16	16	pos
5 (PO1068)	-	-	pos
6 (PO1069)	16	16	pos
7 (PO1070)	70	-	pos
8 (PO1072)	52/33/35/58	52	pos
9 (PO1075)	not valid	58	pos
10 (PO1080)	52/33/35/58, 31	31,52	pos
11 (PO1081)	82	82	pos
12 (PO1082)	16, 62	16	pos
13 (PO1084)	-	56	pos
14 (PO1060)	-	-	neg
15 (PO1061)	-	-	neg
16 (PO1071)	-	-	neg

Detected HPV types are indicated by numbers for Linear Array and LDR.

## Discussion

Testing for hrHPV DNA in primary screening, cytology triage and follow-up of treated patients has gained widespread acceptance as it has been shown to increase the accuracy of screening [8], [9], [38]. Oligonucleotide microarrays in glass slide [14], [16], [39] and Luminex suspension bead array [40] platforms have been previously shown to be able to detect HPV PCR products in a type-specific manner. Importantly, Delrio-Lafreniere and co-workers [13] earlier established a HPV microarray similar to ours. Both tests use the same region for original amplification, but the PCR primer and LDR probe sequences are different and our test detects a higher number of HPV genotypes. Also, single nucleotide extension based microarray detection (APEX) of HPV 16 E6 region was reported by Gemignani and coworkers, showing the capability for highly specific HPV 16 variant discrimination [41].. APEX has been used for detection of multiple HPV types as well [42]. In this study we have developed a microarray assay based on improved PGMY multiplex PCR primers and ligation reaction for the detection of clinically important hrHPV types together with two common low risk HPV types. Additionally, to improve the accuracy of microarray read-out, we have included a hybridization control probe to enable normalization of background and technical variation between spots [32].

Specific LDR probes were found for all 15 hrHPV types and two lrHPV types included in the study, showing good overall performance of our test using plasmid templates. However, design of new probes to replace non-functional ones is needed for the detection of three lrHPV types (HPVs 11, 43, 44) and for those types with only one functional probe. It should be emphasized that each probe requires individual experimental validation because current tools do not allow reliable testing of probe candidate functionality on the basis of *in silico* alignment to target genomic sequences. Furthermore, we found the multiplexing capacity of our PGMY-t primer set superior to other tested primer sets. This is in agreement with Gheit and coworkers, who reported that type-specific multiplex PCR primer pool performs better than GP5+/6+ primers [42]. Generally, the performance of our PGMY-t primers was similar to that of the original PGMY primers but with less variability in microarray signals. A possible disadvantage of our PGMY-t primer set, similar to other generally used HPV PCR amplification systems, is that it amplifies the L1 region which may infrequently be lost upon integration of viral DNA into the host genome. However, the E6/E7 region is always retained [44] and consequently may be a more sensitive marker of infection than the L1 region [20].

For any test in routine diagnostic use, an objective standard for target presence or absence is necessary to eliminate problems of interpretation. Typically these criteria have not been discussed in reports of diagnostic HPV microarrays but instead obtained signals are interpreted more or less visually as a sign of presence of a given type [14], [16], [41]–[43]. The approach we have adopted here is based on testing a set of known positive and negative signals against a distribution of negative signals collected from multiple specificity experiments. Since the background signal distribution and the test data were collected from several microarrays and probe-template combinations, they include the array-to-array variation as well as the probe-to-probe variation in ligation and hybridization.

Studies comparing a HPV DNA microarray with other detection methods like sequencing [14] or Hybrid Capture 2 and Linear array HPV genotyping [45] have reported equal or better clinical sensitivity for microarray. This is in accordance with our findings with a limited number of patient samples where our LDR microarray identified multiple hrHPV types in samples remaining hrHPV negative by HC2 assay. The LDR microarray was highly consistent in identifying multiple HPV types when the same patient sample was tested in triplicate or quadruplicate, and the results were further confirmed by sequencing the PCR amplicons, suggesting reliable genotyping of patient samples. Comparison of our LDR test with Linear Array and HC2 using a second set of patient samples further suggested that the performance of LDR in HPV genotyping is as good as that of LA. However, LA requires more manual labor and relies on visual inspection of results. LDR in contrast can be automated to further improve the throughput of the assay. Our test is also more flexible than other microarrays because new targets can be easily added to an existing microarray platform. The platform can be further expanded to detect other viruses or other clinically relevant pathogens.

In conclusion, we have demonstrated sensitive and accurate HPV genotyping by type-specific multiplex PCR followed by ligation reaction and microarray detection, together with objective criteria for the presence or absence of HPV types. Preliminary evaluation demonstrates the feasibility of our test for clinical samples. In future, HPV genotyping is likely to be launched to clinical use for the management and follow-up of patients with cervical epithelial abnormalities requiring accurate and economical high-throughput testing.

## Supporting Information

File S1 **Sequences of PCR primers, LDR probes and ZipCodes.**
(XLS)

File S2 **Patient sample p403-2z triplicate.** The HPV probe pool signals in three hybridizations of sample p403-2z and associated p-values are shown.
(PDF)

File S3 **Sequencing results of two different patient samples with HPV type-specific primers.** The used primers are the same as in PGMY-t multiplex pool. The files are AB1 trace files.
(TAR)

File S4 **Comparison of PGMY-t and original PGMY multiplex primer systems at 100 pg and 10 pg template concentrations.** This figure complements data presented in Figure 3.
(PDF)

File S5 **Results of dilution series experiment using HPV 16 and 18 plasmids.** Boxplots showing the signals of HPV 16 and 18 HPV LDR probes and other HPV LDR probes.
(PDF)

File S6 **Linear Array results of 16 patient samples.** The figure shows scanned pictures of each LA genotyping strip with reference guide for 16 patient samples.
(PDF)

File S7 **LDR results of 16 patient samples.** The figure shows boxplots of each HPV LDR probe for 16 patient samples that were also analyzed with LA and HC2 (see Table 2).
(PDF)
