# Supplementary material for: Detection of Human Papillomaviruses by Polymerase Chain Reaction and Ligation Reaction on Universal Microarray
Source: PLoS One. 2012 Mar 23;7(3):e34211. doi: 10.1371/journal.pone.0034211 (PMC3311614; doi:10.1371/journal.pone.0034211)
Supplement: File S2 — Patient sample p403-2z triplicate. The HPV probe pool signals in three hybridizations of sample p403-2z and associated p-values are shown. (PDF) [file pone.0034211.s002.pdf]

Patient sample p403/2z, microarray 1

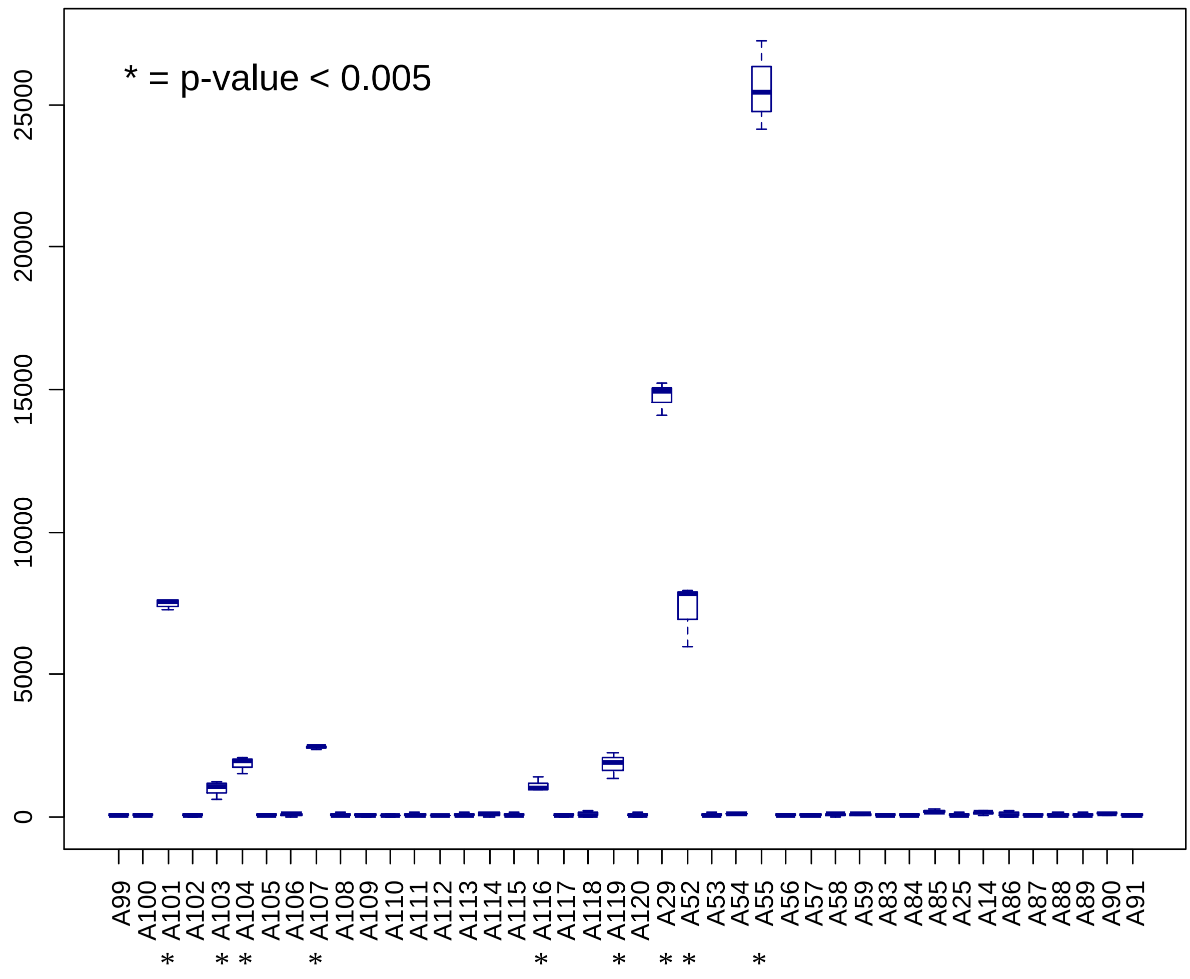

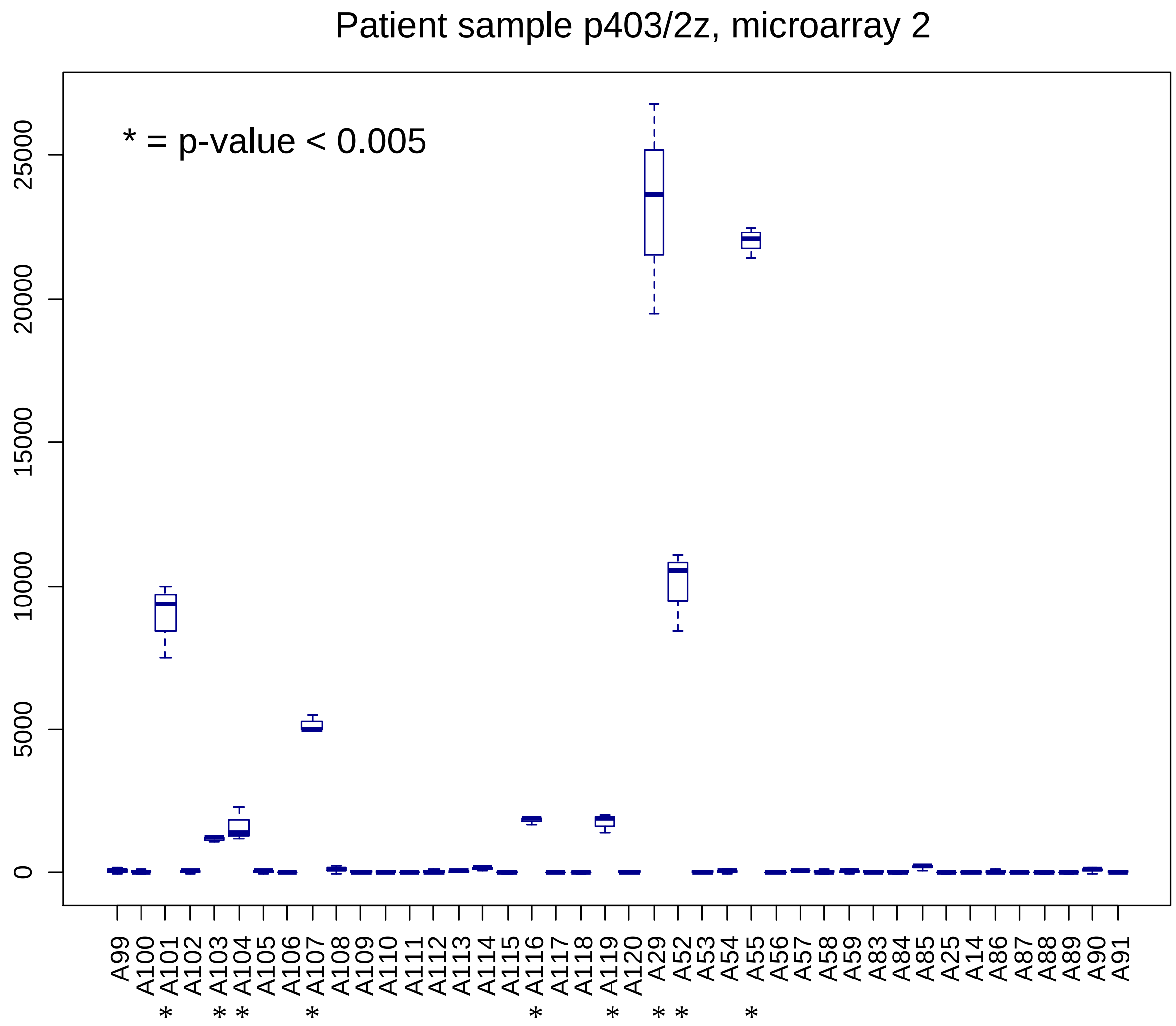

Patient sample p403/2z, microarray 2
* = p-value < 0.005
0
5000
10000
15000
20000
25000
A99 A100 A101 A102 A103 A104 A105 A106 A107 A108 A109 A110 A111 A112 A113 A114 A115 A116 A117 A118 A119 A120 A29 A52 A53 A54 A55 A56 A57 A58 A59 A83 A84 A85 A25 A14 A86 A87 A88 A89 A90 A91

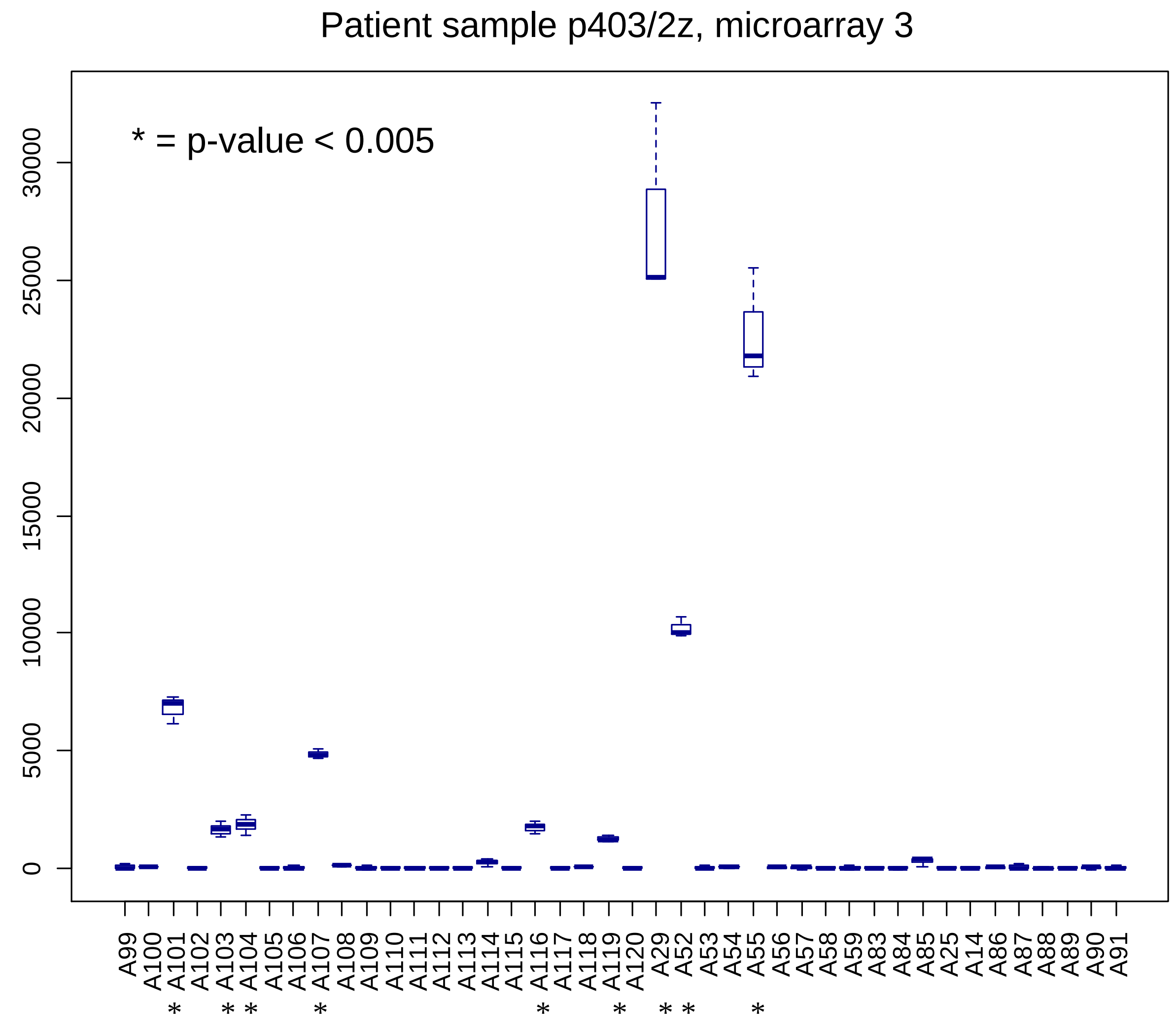
Patient sample p403/2z, microarray 3
* = p-value < 0.005
0
5000
10000
15000
20000
25000
30000
A99 A100 A101 A102 A103 A104 A105 A106 A107 A108 A109 A110 A111 A112 A113 A114 A115 A116 A117 A118 A119 A120 A29 A52 A53 A54 A55 A56 A57 A58 A59 A83 A84 A85 A25 A14 A86 A87 A88 A89 A90 A91
* * * * * * * * *

Mann-Whitney P-values of each probe on each microarray.

ZipCode	array 1	array 2	array 3
		p-value	
A99	0.9972	0.8127	0.8667
A100	0.9982	0.9901	0.9643
*A101	0.0014	0.0014	0.0014
A102	0.9925	0.9919	0.9964
*A103	0.0015	0.0014	0.0014
*A104	0.0014	0.0014	0.0014
A105	0.9984	0.9847	0.9855
A106	0.9302	0.9983	0.9892
*A107	0.0014	0.0014	0.0014
A108	0.8884	0.3917	0.0708
A109	0.9924	0.9979	0.9104
A110	0.9982	0.9977	0.9928
A111	0.9974	0.9959	0.9951
A112	0.9967	0.9681	0.9608
A113	0.9962	0.9900	0.9957
A114	0.8695	0.1424	0.0529
A115	0.9953	0.9980	0.9970
*A116	0.0014	0.0014	0.0014
A117	0.9961	0.9983	0.9925
A118	0.8460	0.9970	0.7032
*A119	0.0014	0.0014	0.0014
A120	0.9960	0.9970	0.9972
*A29	0.0014	0.0014	0.0014
*A52	0.0014	0.0014	0.0014
A53	0.9819	0.9951	0.9009
A54	0.9729	0.9682	0.8263
*A55	0.0014	0.0014	0.0014
A56	0.9983	0.9977	0.8626
A57	0.9984	0.9888	0.9413
A58	0.9286	0.9952	0.9981
A59	0.9501	0.9749	0.9116
A83	0.9954	0.9963	0.9966
A84	0.9943	0.9960	0.9961
A85	0.0986	0.1391	0.0338
A25	0.9921	0.9978	0.9967
A14	0.3676	0.9969	0.9919
A86	0.9245	0.9981	0.9781
A87	0.9985	0.9979	0.9537
A88	0.9974	0.9978	0.9977
A89	0.9963	0.9968	0.9906
A90	0.8341	0.6045	0.8756
A91	0.9972	0.9979	0.9979
