# Supplementary material for: Detection of Human Papillomaviruses by Polymerase Chain Reaction and Ligation Reaction on Universal Microarray
Source: PLoS One. 2012 Mar 23;7(3):e34211. doi: 10.1371/journal.pone.0034211 (PMC3311614; doi:10.1371/journal.pone.0034211)
Supplement: File S4 — Comparison of PGMY-t and original PGMY multiplex primer systems at 100 pg and 10 pg template concentrations. This figure complements data presented in Figure 3. (PDF) [file pone.0034211.s004.pdf]

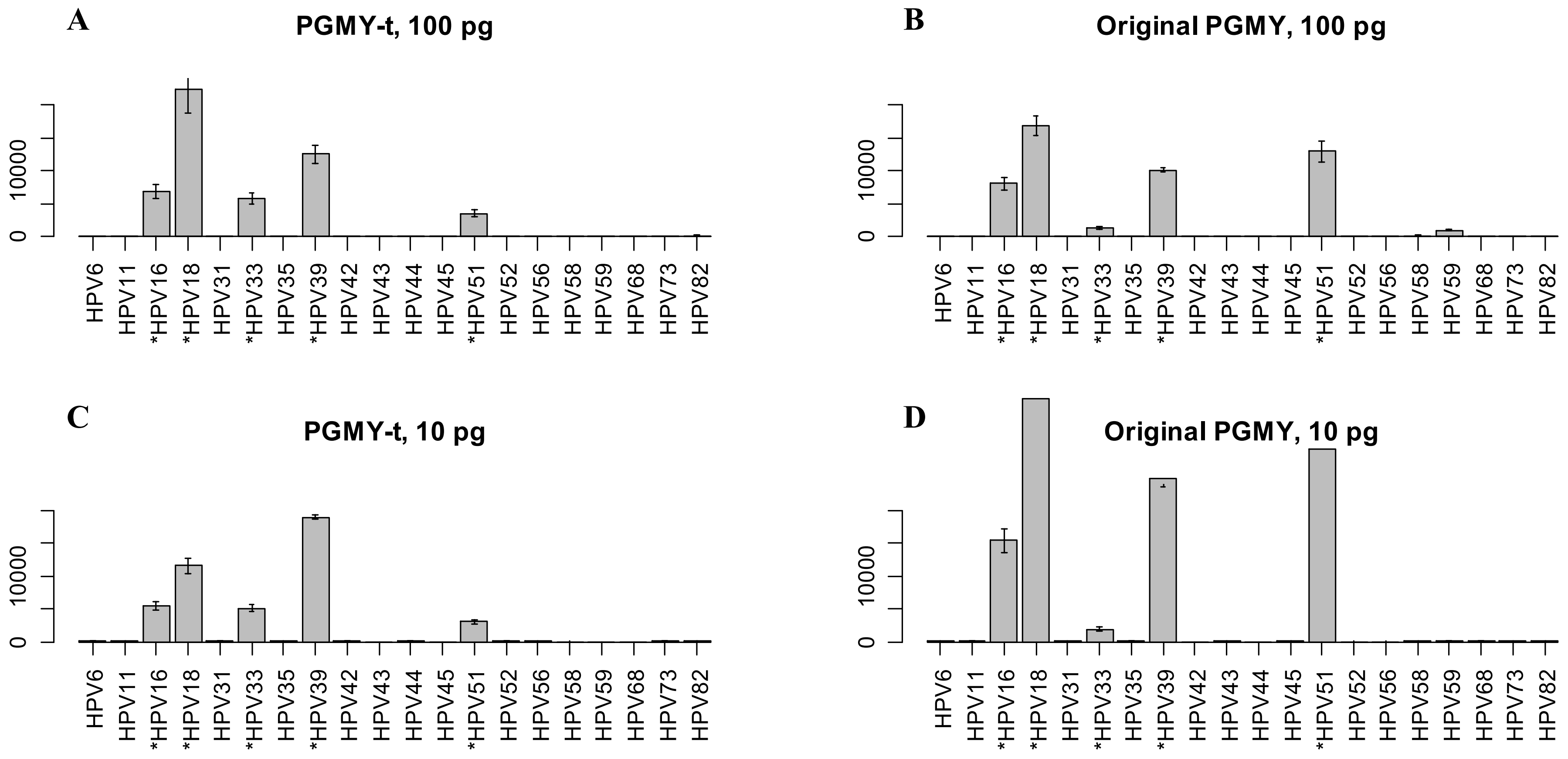

Comparison of PGMY-t and the original PGMY multiplex PCR primer mixes at 100 pg and 10 pg template concentrations. Both primer mixes at 0.2 μM primer concentration amplify all five HPV types from 100 pg of template (A) & (B) and from 10 pg of template (C) & (D). With the original PGMY mix at 100 pg template concentration, HPV 59 gives a false positive signal (B). Data are presented as means±SD from two independent microarrays. Y-axis shows signal intensity in arbitrary units. Asterisk (*) indicates the target HPV types present in the experiment.
