# Supplementary material for: Detection of Human Papillomaviruses by Polymerase Chain Reaction and Ligation Reaction on Universal Microarray
Source: PLoS One. 2012 Mar 23;7(3):e34211. doi: 10.1371/journal.pone.0034211 (PMC3311614; doi:10.1371/journal.pone.0034211)
Supplement: File S5 — Results of dilution series experiment using HPV 16 and 18 plasmids. Boxplots showing the signals of HPV 16 and 18 HPV LDR probes and other HPV LDR probes. (PDF) [file pone.0034211.s005.pdf]

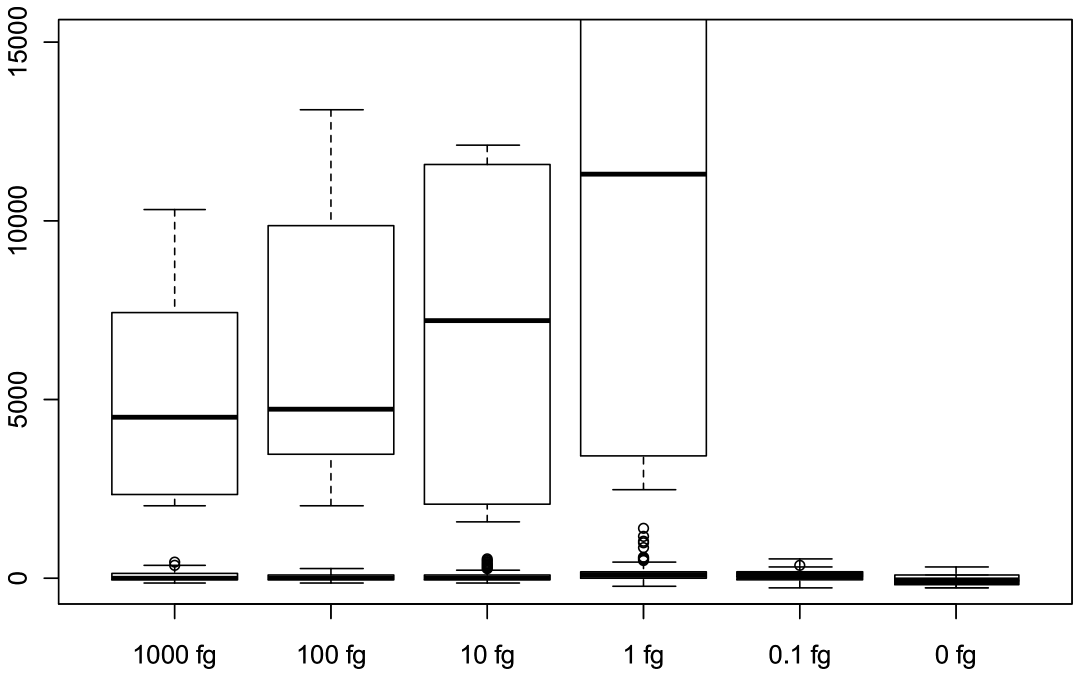

Dilution series of HPV16 and 18 plasmid PCR templates at concentrations from 1000 fg to 0 fg. The PCR amplicons were detected by LDR on microarray. The upper boxplots represent microarray signals from HPV16 and 18 LDR probes. The lower boxplots represent signals from oher HPV LDR probes.
