# Supplementary material for: Detection of Human Papillomaviruses by Polymerase Chain Reaction and Ligation Reaction on Universal Microarray
Source: PLoS One. 2012 Mar 23;7(3):e34211. doi: 10.1371/journal.pone.0034211 (PMC3311614; doi:10.1371/journal.pone.0034211)
Supplement: File S6 — Linear Array results of 16 patient samples. The figure shows scanned pictures of each LA genotyping strip with reference guide for 16 patient samples. (PDF) [file pone.0034211.s006.pdf]

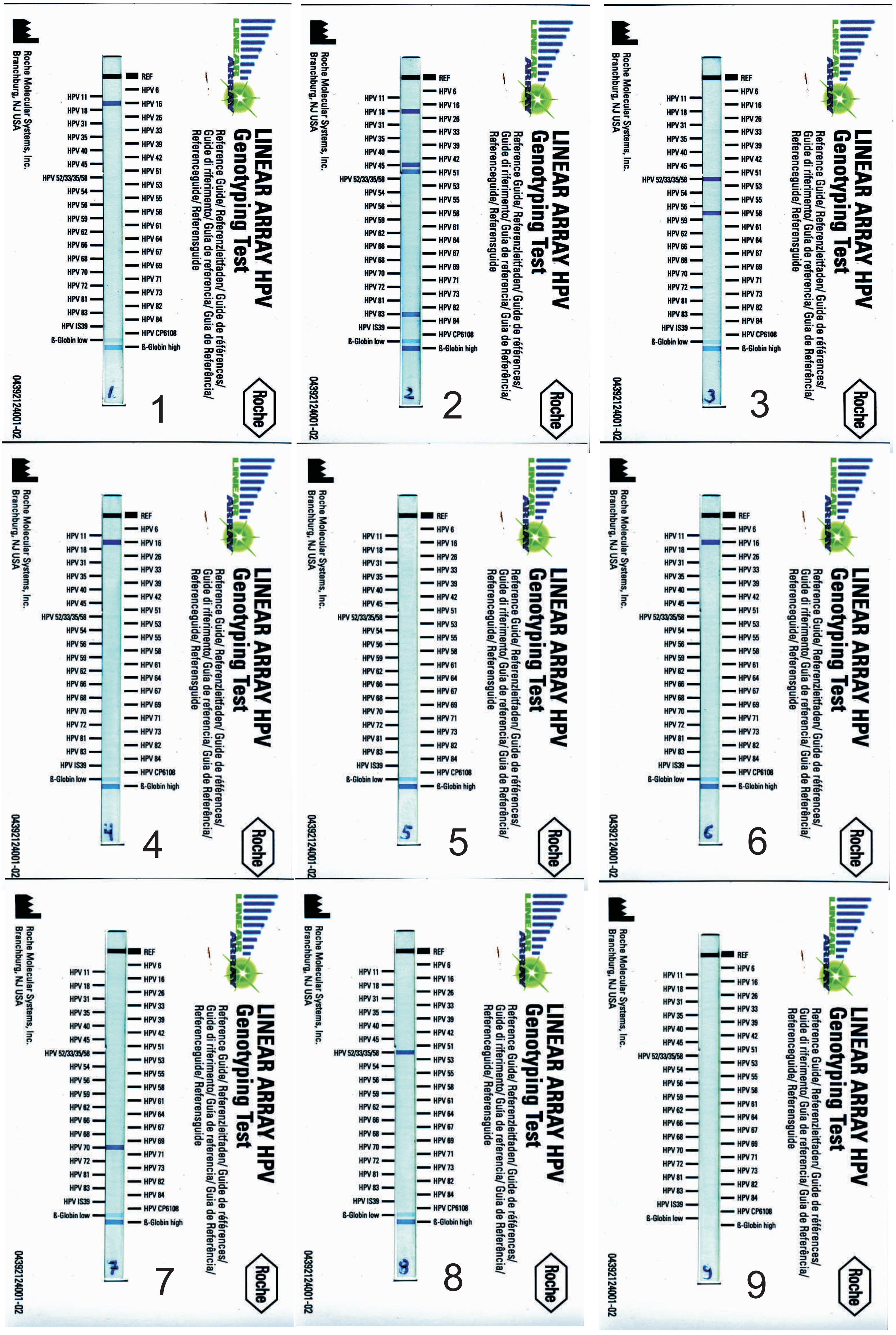
LINEAR ARRAY HPV Genotyping Test
Reference Guide/ Referenzleitfaden/ Guide de références/ Guida di riferimento/ Guía de referencia/ Guia de Referência/ Referenceguide/ Referensguide
Roche Molecular Systems, Inc. Branchburg, NJ USA
Roche
04392124001-02
REF
HPV 6
HPV 16
HPV 26
HPV 33
HPV 39
HPV 42
HPV 51
HPV 53
HPV 55
HPV 58
HPV 61
HPV 64
HPV 67
HPV 69
HPV 71
HPV 73
HPV 82
HPV 84
HPV CP6108
ß-Globin high
HPV 11
HPV 18
HPV 31
HPV 35
HPV 40
HPV 45
HPV 52/33/35/58
HPV 54
HPV 56
HPV 59
HPV 62
HPV 66
HPV 68
HPV 70
HPV 72
HPV 81
HPV 83
HPV IS39
ß-Globin low
1
2
3
4
5
6
7
8
9

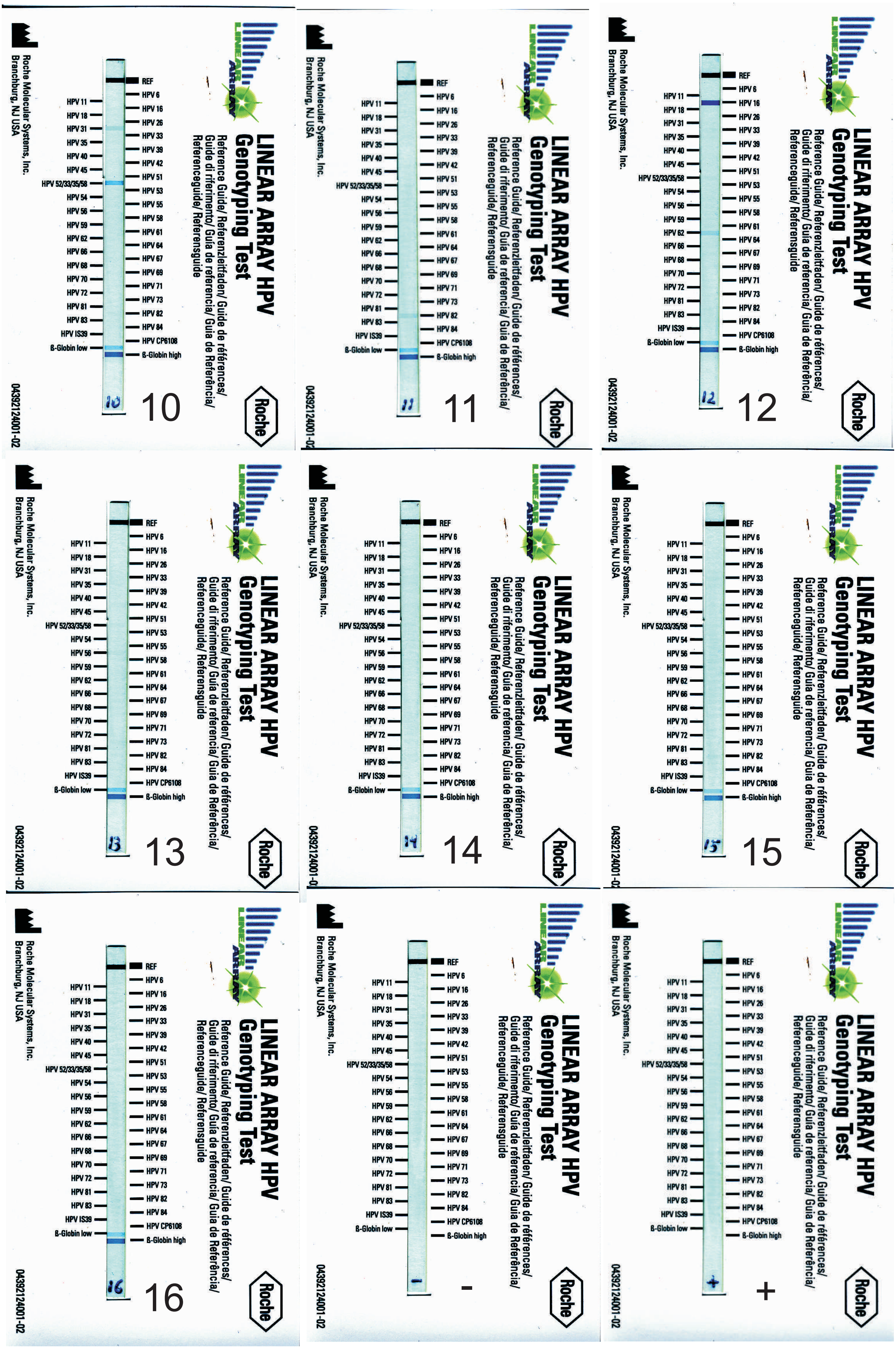
LINEAR ARRAY HPV Genotyping Test
Reference Guide/ Referenzleitfaden/ Guide de références/ Guide di riferimento/ Guía de referencia/ Guia de Referência/ Referenceguide/ Referensguide
Roche Molecular Systems, Inc. Branchburg, NJ USA
Roche
04392124001-02
REF
HPV 6
HPV 11
HPV 16
HPV 18
HPV 26
HPV 31
HPV 33
HPV 35
HPV 39
HPV 40
HPV 42
HPV 45
HPV 51
HPV 52/33/35/58
HPV 53
HPV 54
HPV 55
HPV 56
HPV 58
HPV 59
HPV 61
HPV 62
HPV 64
HPV 66
HPV 67
HPV 68
HPV 69
HPV 70
HPV 71
HPV 72
HPV 73
HPV 81
HPV 82
HPV 83
HPV 84
HPV IS39
HPV CP6108
ß-Globin low
ß-Globin high
10
11
12
13
14
15
16
-
+
