# Supplementary figures and images for: Detection of Human Papillomaviruses by Polymerase Chain Reaction and Ligation Reaction on Universal Microarray
Source: PLoS One. 2012 Mar 23;7(3):e34211. doi: 10.1371/journal.pone.0034211 (PMC3311614; doi:10.1371/journal.pone.0034211)

1 (PO1064)

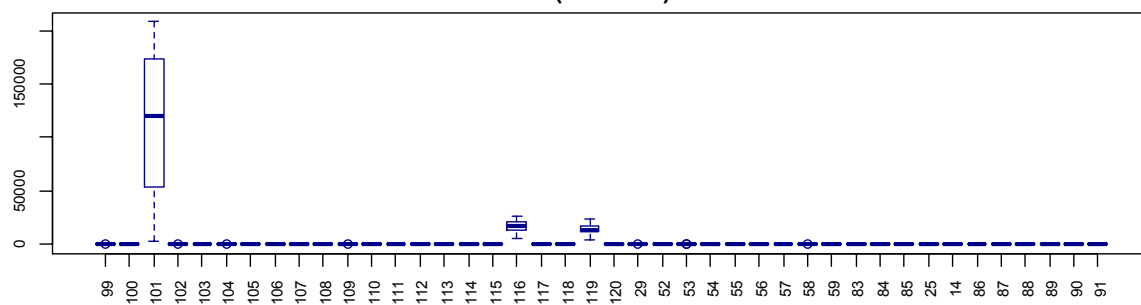

2 (PO1065)

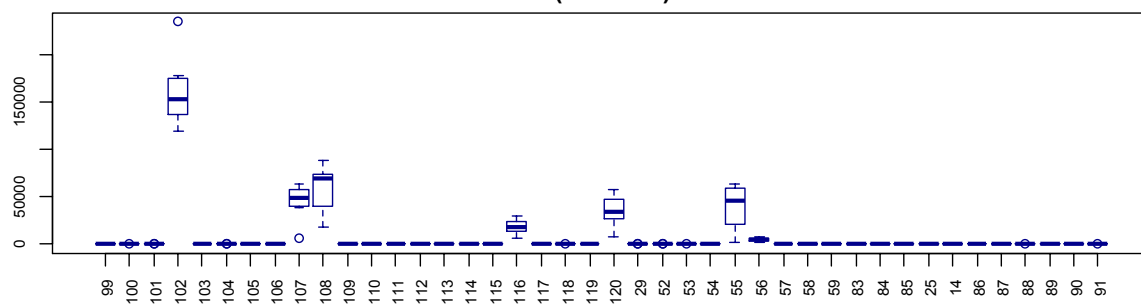

3 (PO1066)

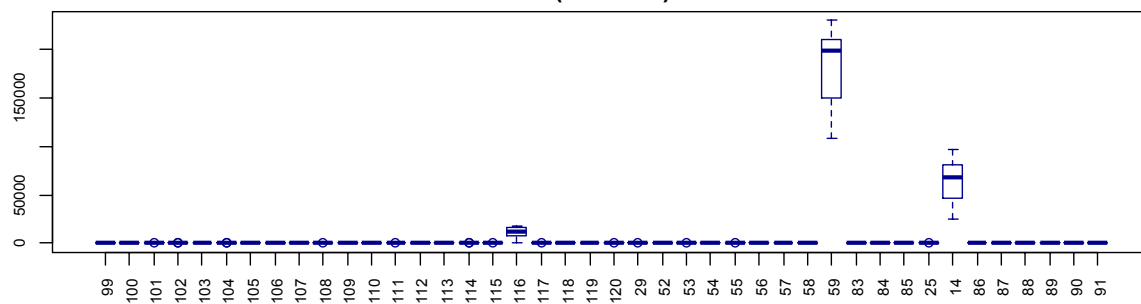

4 (PO1067)

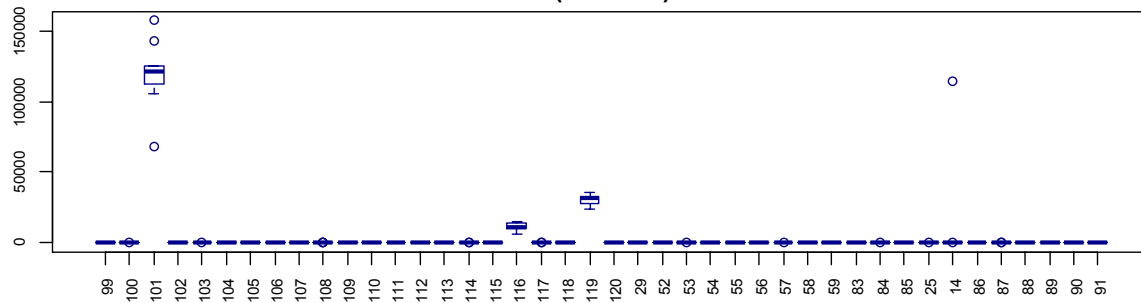

5 (PO1068)

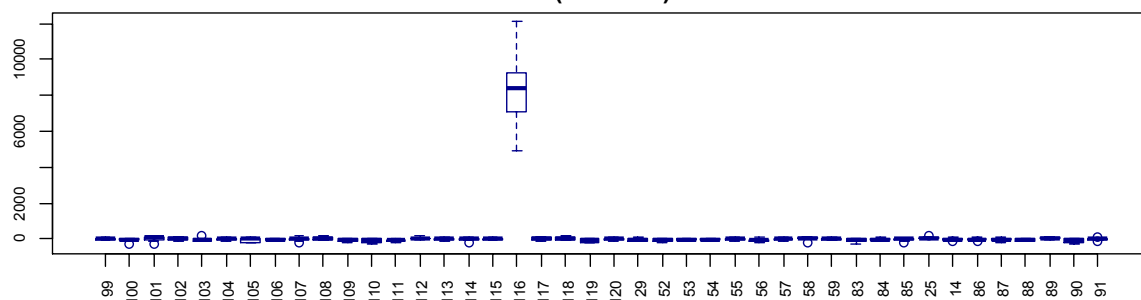

6 (PO1069)

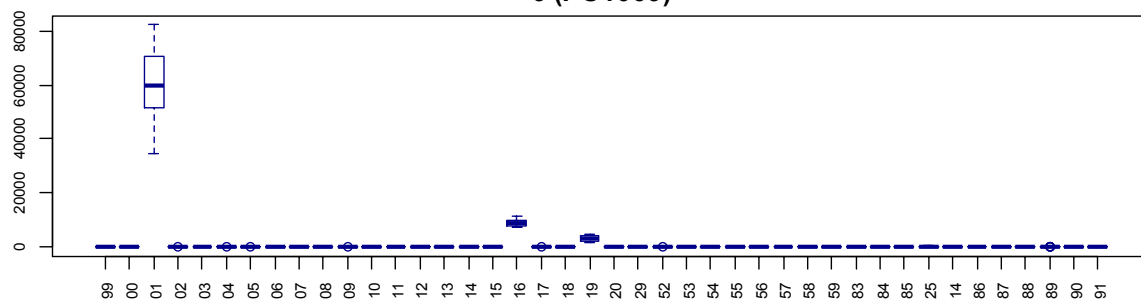

7 (PO1070)

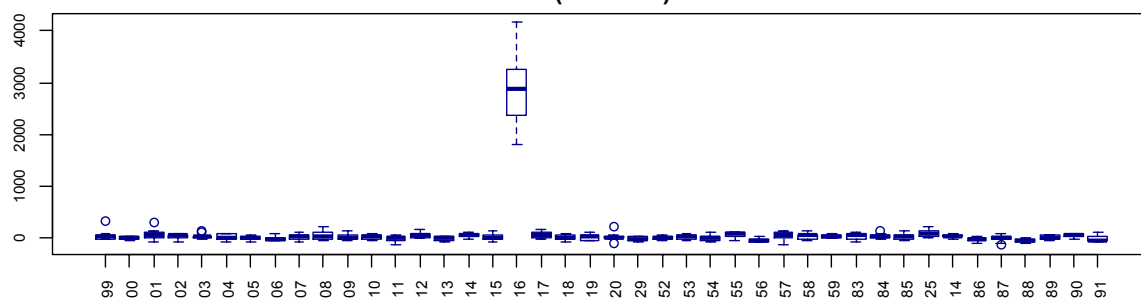

8 (PO1072)

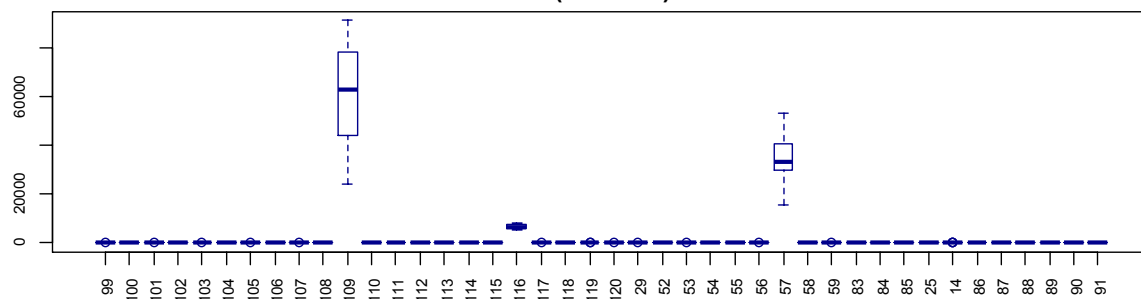

9 (PO1075)

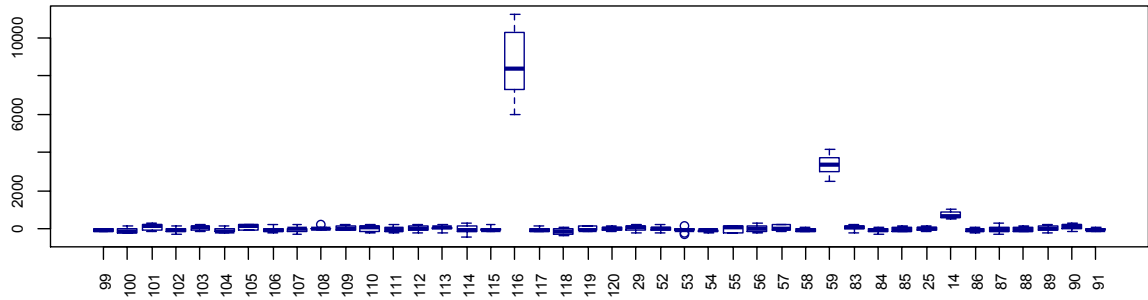

10 (PO1080)

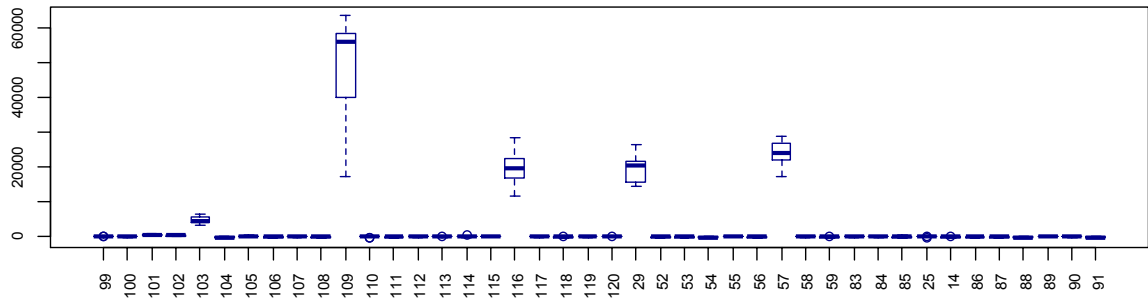

11 (PO1081)

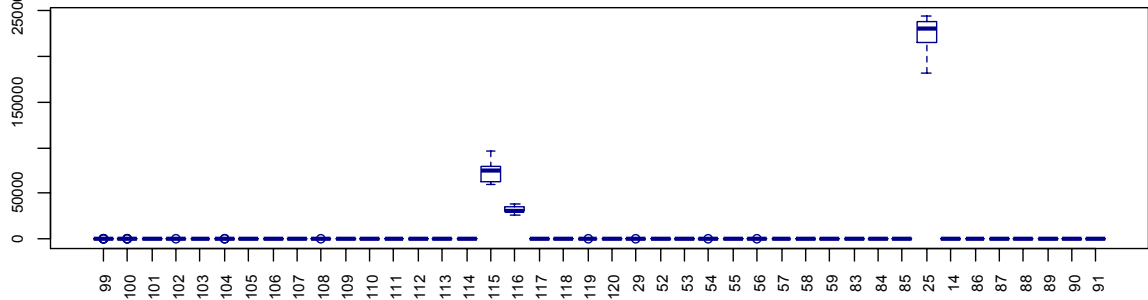

12 (PO1082)

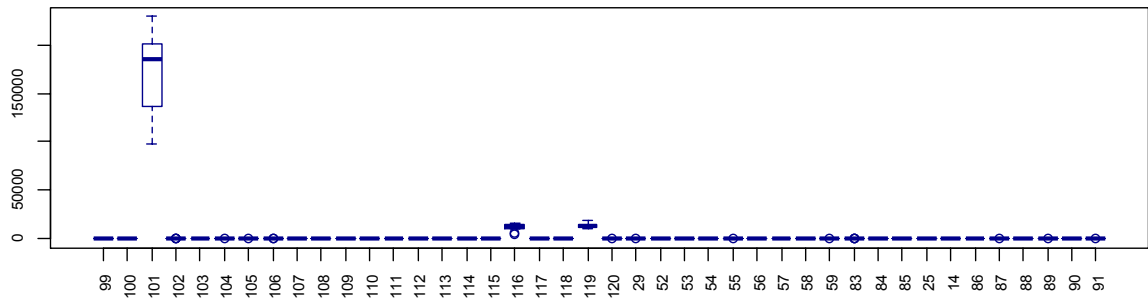

13 (PO1084)

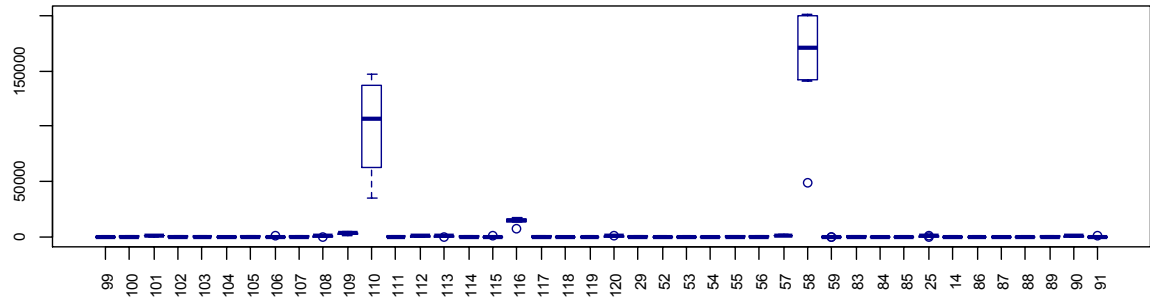

14 (PO1060)

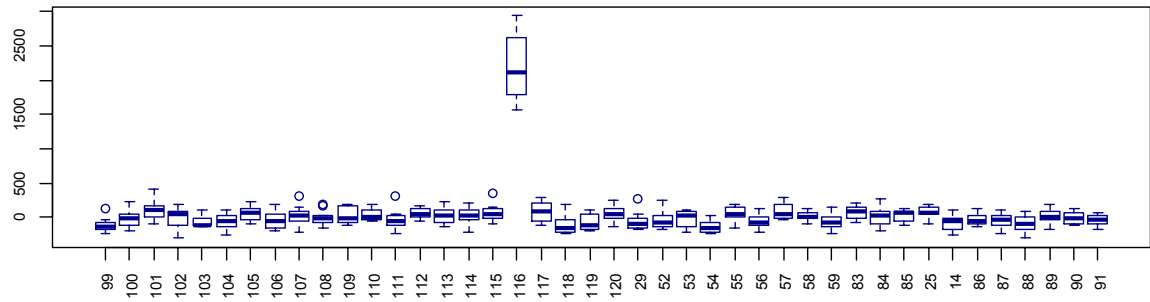

15 (PO1061)

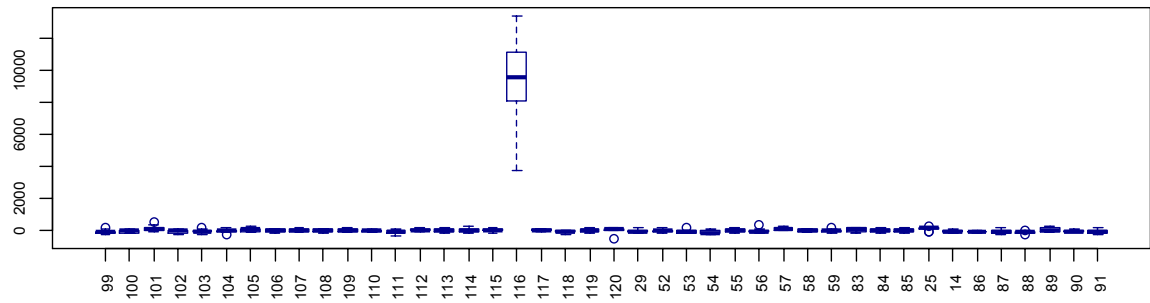

16 (PO1071)

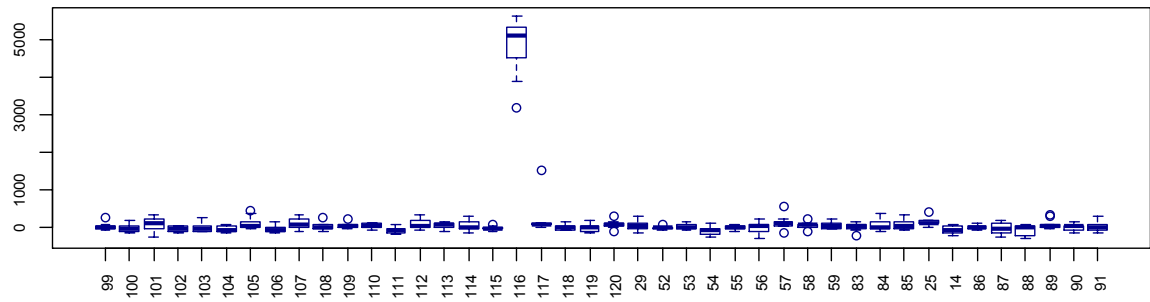

Supplement: File S7 — LDR results of 16 patient samples. The figure shows boxplots of each HPV LDR probe for 16 patient samples that were also analyzed with LA and HC2 (see Table 2). (PDF) [file pone.0034211.s007.pdf]
